# Allogeneic Mesenchymal Stem Cells in Combination with Hyaluronic Acid for the Treatment of Osteoarthritis in Rabbits

**DOI:** 10.1371/journal.pone.0149835

**Published:** 2016-02-25

**Authors:** En-Rung Chiang, Hsiao-Li Ma, Jung-Pan Wang, Chien-Lin Liu, Tain-Hsiung Chen, Shih-Chieh Hung

**Affiliations:** 1 Institute of Clinical Medicine, National Yang-Ming University, Taipei 112, Taiwan; 2 Institute of Pharmacology, National Yang-Ming University, Taipei 112, Taiwan; 3 Department of Surgery, School of Medicine, National Yang-Ming University, Taipei 112, Taiwan; 4 Orthopaedics and Traumatology, Taipei Veterans General Hospital, Taipei 112, Taiwan; 5 Institute of Biomedical Sciences, Academia Sinica, Taipei 115, Taiwan; 6 Integrative Stem Cell Center & Department of Orthopedics, China Medical University Hospital, Taichung 404, Taiwan; 7 Institute of Clinical Medicine, China Medical University, Taichung 404, Taiwan; University of Wisconsin-Madison, UNITED STATES

## Abstract

Mesenchymal stem cell (MSC)-based therapies may aid in the repair of articular cartilage defects. The purpose of this study was to investigate the effects of intraarticular injection of allogeneic MSCs in an *in vivo* anterior cruciate ligament transection (ACLT) model of osteoarthritis in rabbits. Allogeneic bone marrow-derived MSCs were isolated and cultured under hypoxia (1% O_2_). After 8 weeks following ACLT, MSCs suspended in hyaluronic acid (HA) were injected into the knees, and the contralateral knees were injected with HA alone. Additional controls consisted of a sham operation group as well as an untreated osteoarthritis group. The tissues were analyzed by macroscopic examination as well as histologic and immunohistochemical methods at 6 and 12 weeks post-transplantation. At 6 and 12 weeks, the joint surface showed less cartilage loss and surface abrasion after MSC injection as compared to the tissues receiving HA injection alone. Significantly better histological scores and cartilage content were observed with the MSC transplantation. Furthermore, engraftment of allogenic MSCs were evident in surface cartilage. Thus, injection of the allogeneic MSCs reduced the progression of osteoarthritis *in vivo*.

## Introduction

Up to 10% of the people over 60 years of age have osteoarthritis (OA) [1]. Owing to the limited capacity of articular cartilage for self-repair and the growing aging population, the incidence of OA is increasing in many countries. The current treatment options for OA include the use of anti-inflammatory drugs and lubricating supplements, as well as surgeries, such as drilling, microfracture and mosaicplasty. However, these modalities only transiently improve the symptoms. Cell-based therapy may hold promise for the repair of articular cartilage defects as transplantation of autologous chondrocytes was effective in treating cartilage defects in the knee [2]. Because this approach is limited by the number of healthy chondrocytes in patients, progenitor or stem cells have emerged as an alternative.

Mesenchymal stem cells (MSCs) are capable of self-renewal and differentiating into different mesenchymal tissues, and their use in tissue engineering of cartilage and bone and osteogenesis imperfecta has been reported [3]. Furthermore, the safety of MSCs has been demonstrated in two phase III studies [4]. Although allogeneic MSCs have been applied for regenerative medicine in animal studies and tested in clinical trials, several studies argued that MSCs are not intrinsically immunoprivileged [5] and rejection of allogeneic MSCs has been observed in immunocompetent recipients [6–8]. Therefore, several measures including change of oxygen conditions during culture have been investigated to prevent allogeneic MSCs from rejection by host immune systems [5;9].

Although intra-articular injection of hyaluronic acid (HA) has been employed in OA treatment, its effects on chondroprotection and the prevention of OA progression of the knee remain controversial [10;11]. Although the effect of HA in treating OA remains to be defined, many preclinical [12] and clinical treatment protocols involving cell-based therapy use HA as a vehicle [13]. Use of HA as a vehicle may have the added benefit of ensuring delivery of MSCs to the articular surface given that it localizes to the articular surface after intra-articular infusion.

We hypothesize that MSCs can be applied for treating OA in allogeneic recipients. In the present study, we developed standardized procedures for the isolation and expansion of MSCs under hypoxic condition (1% O_2_). In addition, we compared the therapeutic potential of these MSCs co-injected with HA and HA alone for OA by examining articular cartilage regeneration in an *in vivo* anterior cruciate ligament transection (ACLT) model of OA established in rabbits [14].

## Materials and Methods

### Isolation of rabbit bone marrow MSCs

The study protocol was approved by the institutional animal welfare guidelines of Taipei Veterans General Hospital (Taipei, Taiwan). Both femurs from two skeletally mature New Zealand white rabbits were removed, and the soft tissues were detached aseptically. Bone marrow was extruded by inserting an 18-gauge needle into the shaft of the bone and flushing it with basal medium consisting of α-minimal essential medium (α-MEM; Gibco-BRL, Gaithersburg, MD). Mononuclear cells were isolated from the bone marrow aspirates by the density gradient centrifugation, suspended in complete culture medium (CCM) consisting of α-MEM supplemented with 16.6% fetal bovine serum (FBS), 100 U/mL penicillin, 100 μg/mL streptomycin, and 2mM L-glutamine, and seeded in plastic dishes. After 24 h, nonadherent cells were removed. After 9 days, the cells (passage 0) were harvested for further subculturing. Starting from passage 1, the cells were seeded at 100 cells/cm^2^ in CCM. For hypoxic cultures, cells were cultured in a gas mixture composed of 94% N_2_, 5% CO_2_, and 1% O_2_.

### *In vitro* osteogenesis and adipogenesis of MSCs

MSCs were maintained in one of the following culture conditions: (i) osteogenic differentiation medium consisting of α-MEM supplemented with 10% FBS, 50 g/mL ascorbate-2 phosphate (Nacalai, Kyoto, Japan), 10^-8^M dexamethasone (Sigma, St. Louis, MO) and 10mM β-glycerophosphate (Sigma) or (ii) adipogenic differentiation medium comprised of α-MEM supplemented with 10% FBS, 50 μg/mL ascorbate-2 phosphate, 10^-7^M dexamethasone, 50 μg/mL indomethacin (Sigma), 0.45mM 3-isobutyl-1-methylxanthine (Sigma) and 10 μg/mL insulin (Sigma). The medium was changed every 3 days until the appearance of morphologic features of differentiation, at which time the cells were evaluated using by histochemical and immunofluorescence analyses.

### *In vitro* chondrogenesis of MSCs

At semi-confluence, 5×10^5^ MSCs were trypsinized and centrifuged at 500 ×g for 10 min. Within 12–24 h of incubation in CCM, the cells formed a spherical aggregate. FBS-containing medium was then replaced with chondrogenic medium consisting of serum-free high-glucose Dulbecco’s modified Eagle’s medium supplemented with ITS+Premix (BD Biosciences, Bedford, MA), 6.25 μg/mL insulin, 6.25 μg/mL transferrin, 6.25 μg/mL selenious acid, 1.25 mg/mL bovine serum albumin (BSA), 5.35 mg/mL linoleic acid, 10^−7^M dexamethasone, 50 μg/mL ascobate-2-phosphate and 10 ng/mL TGF-β1 (PeproTech, Rocky Hill, NJ). Medium changes were carried out at 2 to 3-day intervals, and the cell pellets were harvested at 21 days.

### Cell labeling with superparamagnetic iron oxide nanoparticles (SPIO)

SPIO were prepared as described previously [15]. In brief, 0.1M Fe(III) (Sigma) and 0.2M Fe(II) (Sigma) aqueous solutions were prepared by dissolving FeCl_3_ and FeCl_2_, respectively. For production of Fe_3_O_4_ nanoparticles, 4 mL of the Fe(III) and 1 mL of the Fe(II) solutions were mixed at room temperature, and the pH was adjusted to 11 using 5M NaOH. After exposure to a magnet, the precipitates were washed with deionized water after which 3 g of organic acid was added to achieve complete coating of the particle surface. The precipitates were redispersed in deionized water after excess adherents were removed by centrifugation. Before labeling, 50 μg/mL of SPIO was coated by mixing 0.75 μg/mL poly-L-lysine (Sigma) into the culture medium at room temperature for 1 h to facilitate their endocytosis. MSCs were then seeded in a 6-well plate at a density of 4 × 10^4^/ well and cultured for 24 h. The MSCs were incubated in SPIO-containing medium for 24 h and thoroughly washed with PBS.

### ACLT osteoarthritis model

**Fig 1:**
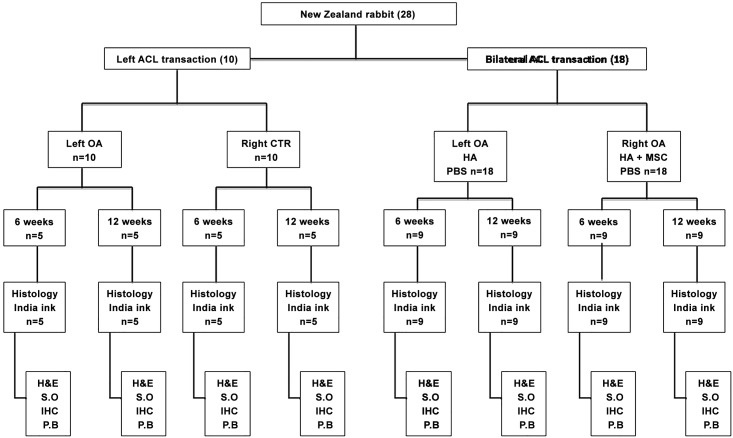
Study design. **Evaluation of the effect of hypoxia-cultured MSCs for treatment of osteoarthritis in rabbits.** The numbers in parentheses indicate number of rabbits receiving ACLT and analysis. CTR: control group; H&E: haematoxylin and eosin; S.O: safranin O; P.B: Prussian blue.

The experiments began in 2012. The knee joints of New Zealand Rabbits 9 months of age (~3.0 kg; range: 2.8–3.5 kg) were divided into the following four groups (Fig 1): (i) OA (OA induction without treatment; n = 10), (ii) contralateral control (sham operation; n = 10), (iii) OA+HA (n = 18), and (iv) contralateral OA+HA+MSCs (n = 18). OA of the knee joints was induced as previously decribed [14]. In brief, a medial arthrotomy was performed. With the knee positioned in full flexion, the patella was dislocated laterally, and the anterior cruciate ligament (ACL) was transected. The wounds were closed and covered with a local antibiotic ointment. All rabbits were returned to their cages after the operation and were allowed to move freely, and 0.2 mg/kg/day of intramuscular Meloxicam was administered for 5 days for pain relief. At 8 weeks after ACLT, the knee joints of the OA+HA group were injected with 0.4 mL (10 mg/mL) high molecular weight HA (Hya-Joint, SciVision Biotech, Taipei, Taiwan), while those of the OA+HA+MSCs were injected with 10^6^ MSCs (passage 1) in 0.4 mL of HA. Half of the rabbits in each group were sacrificed by sedation with pentobarbital (3%) 15–40mg/kg and then CO2 inhalation at 6 weeks, and the other half were sacrificed at 12 weeks post HA or HA plus MSCs treatment.

### Macroscopic examination

The surface of distal femur and proximal tibia were exposed and examined macroscopically. Two mL of India ink was injected onto the tibial plateau with a syringe. After 2 min, the surface was washed with saline, and the staining pattern of the tibial plateau was examined macroscopically.

### Histologic analysis

After the cells were washed twice with PBS, they were fixed in 3.7% paraformaldehyde for 10 min at room temperature and washed twice with PBS. Cells cultured in osteogenic medium were stained for alkaline phosphatase activity; those cultured in adipogenic and chondrogenic media were stained with oil red-O and alcian blue, respectively.

For the *in vivo* study, the femoral condyles with articular cartilage were collected and fixed with 10% neutral buffered formalin (Tonar Biotech; Taipei, Taiwan). The samples were then decalcified in 10% formic acid (Sigma) in PBS (Gibco/BRL, Grand Island, NY). The decalcified femur articular samples were embedded in paraffin, and 4-μm sections in the sagittal plane were prepared. Paraffin-embedded sections were stained with haematoxylin and eosin (HE). Glycosaminoglycan was stained with Safranin O fast green (1% Safranin O) and counterstained with 0.75% hematoxylin, and the total and red-stained areas. The ratio of red-stained area to total area (red:total) in the articular cartilage of each proximal tibia were measured using Image-Pro Plus software, version 5.0.

Prussian blue staining was performed to localize the iron particles in SPIO labeled MSCs or rabbit knee joints that were treated with SPIO-labeled MSCs. For *in vitro* cell staining, the SPIO-labeled cells were washed twice with PBS and fixed with 4% glutaraldehyde, and incubated for 30 min with 2% potassium ferrocyanide in 6% hydrochloric acid. After washing three times with PBS, the iron content of cells was examined. For *in vivo* cell tracking, paraffin-embedded sections were deparaffinized and hydrated by distilled water. The sections were then immersed in equal parts of 12% hydrochloric acid and 4% potassium ferrocyanide (Sigma) for 30 min. After washing the sections in distilled water three times, the sections were counterstained with nuclear fast red (Sigma) for 5 min and rinsed twice in distilled water.

### Immunohistochemistry analysis

After the femur articular sections were rehydrated, endogenous peroxidase was blocked with 3% hydrogen peroxide (Sigma). Type II collagen was retrieved with a mixture of 2.5% hyaluronidase (Sigma) and 1 mg/mL of Pronase in PBS (pH 7.4; Sigma) at 37°C for 1 h; type X collagen was retrieved by treatment with 0.1 units/mL of chondroitinase ABC (Sigma) at 37°C for 1 h, followed by treatment with 1 mg/mL of pepsin (Sigma) in Tris HCl (pH 3.0, MDBio, Taipei, Taiwan) at 37°C for 15 min. Sections were then blocked with Ultra V block (Thermo Scientific, Fremont, CA) for 10 min and incubated with primary antibodies against type II collagen (mouse monoclonal antibody; 1:200; CP18; Calbiochem, La Jolla, CA) and type X collagen (rat polyclonal antibody; 1:200; ab58632; Abcam, Cambridge, MA) at 37°C for 4 h. The secondary antibodies were incubated for 30 min using biotin-labeled goat anti-mouse immunoglobulin for type II collagen (Dako, Carpinteria, CA) and biotin-labeled goat anti-rabbit immunoglobulin for type X collagen (Biocare Medical, Walnut Creek, CA), and horseradish peroxidase–conjugated streptavidin (Biocare Medical). After the sections were stained with a 3,3-diaminobenzidine solution containing 0.01% hydrogen peroxide, they were counterstained with hematoxylin (Sigma). The ratio of type II and X collagen stained areas to total area in the articular cartilage of each femur were measured using Image-Pro Plus software, version 5.0.

### Modified Mankin score

The level of articular cartilage degeneration in the knees was evaluated using the modified Mankin score [16], that includes the following four variables: (i) surface (0 = normal, 1 = irregular, 2 = fibrillation or vacuoles, and 3 = blisters or erosion), (ii) hypocellularity (0 = normal, 1 = small decrease in chondrocytes, 2 = large decrease in chondrocytes, and 3 = no cells), (iii) clones (0 = normal, 1 = occasional duos, 3 = duos or trios, and 3 = multiple nested cells), and (iv) alcianophilia (0 = normal, 1 = small decrease in color, 2 = large decrease in color, and 3 = no color). The higher the score, the greater the level of OA. The entire histological evaluation was performed by three investigators who were blinded to the group allocation.

### Statistical analysis

All data are expressed as mean and standard deviation (SD). Statistical comparisons of the histopathological grade among the four groups were performed with non-parametric tests, such as the Wilcoxon test. Differences were considered significant when the p-value was < 0.05. All statistical analyses were conducted using SPSS version 11.0 (SPSS Inc., Chicago, IL).

## Results

### Differentiation potential of hypoxia-treated rabbit MSCs

To examine whether exposure to hypoxia alters the multi-potent differentiation ability of MSCs, cells were cultured in different differentiation mediums. Representative photomicrographs showed that rabbit bone marrow-derived MSCs exposed to hypoxia retained the ability to differentiate into adipogenic lineages, which was verified by the intracellular accumulation of Oil-Red-O-stained lipid vesicles (S1A Fig) as well as osteogenic and chondrogenic lineages as detected by Alizarin Red S and Alcian-blue staining, respectively (S1B and S1C Fig, respectively). These data suggest that hypoxia-treated rabbit MSCs are still multi-potent and can differentiate into bone, fat and cartilage lineage cells.

### Gross appearance of the femur and tibia after injection of the rabbit MSCs

**Fig 2:**
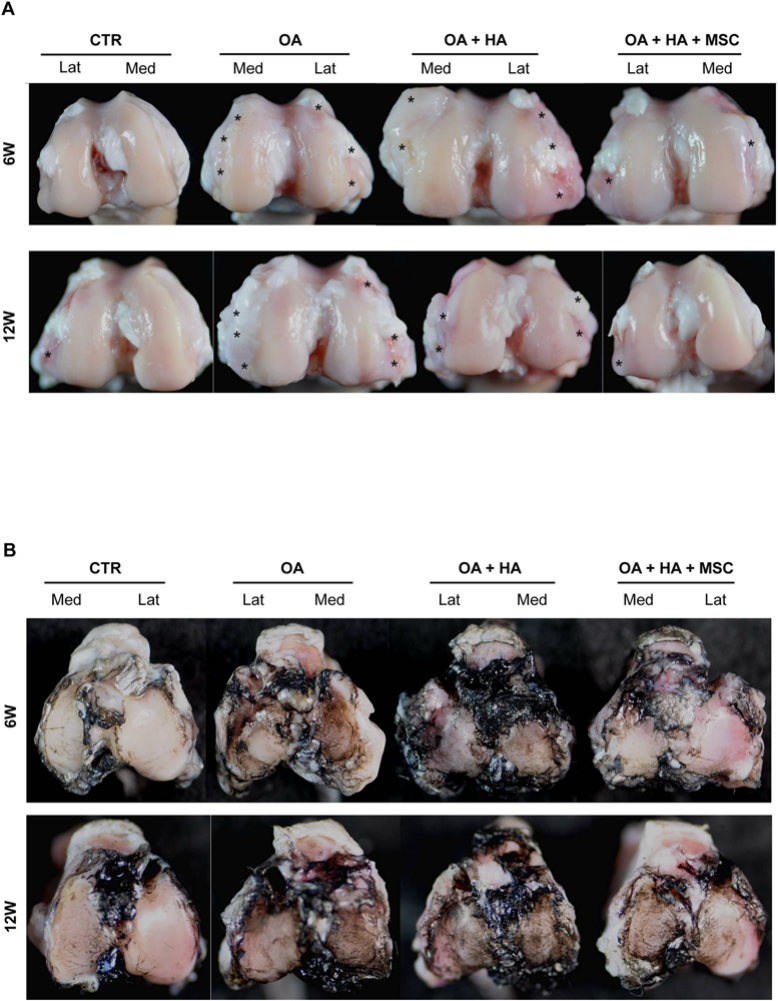
Macroscopic examination of the effects of hypoxia-cultured MSCs on osteoarthritis progression. **(A)** Femur condyles 6 and 12 weeks post-treatment. Asterisks indicate osteophyte formation. **(B)** India ink staining for articular surface of tibial plateaus.

The gross appearance of the knee joints in each group was first evaluated. The joint surface of the knee joints that received ACLT without (OA) or with HA alone (OA+HA) showed marked gross changes of OA, including cartilage abrasion, osteophyte formation (asterisks), and sub-chondral bone exposure (Fig 2A), suggesting HA alone is not effective in suppressing gross OA changes. However, after injection of the MSCs plus HA (OA+HA+MSC), the joint surface showed diminished gross changes of OA. India ink staining, which has been used to detect irregularity of articular cartilage surface, showed more fissures over the tibia condyle located mainly at the medial side in the groups without cell injection (Fig 2B). Treatment with MSCs plus HA reduced the formation of fissures on the surface at both 6 and 12 weeks of post-treatment. These data suggest that MSCs plus HA is better than HA alone in suppressing gross OA changes.

### HE and Safranin-O staining of the femur condyles

**Fig 3:**
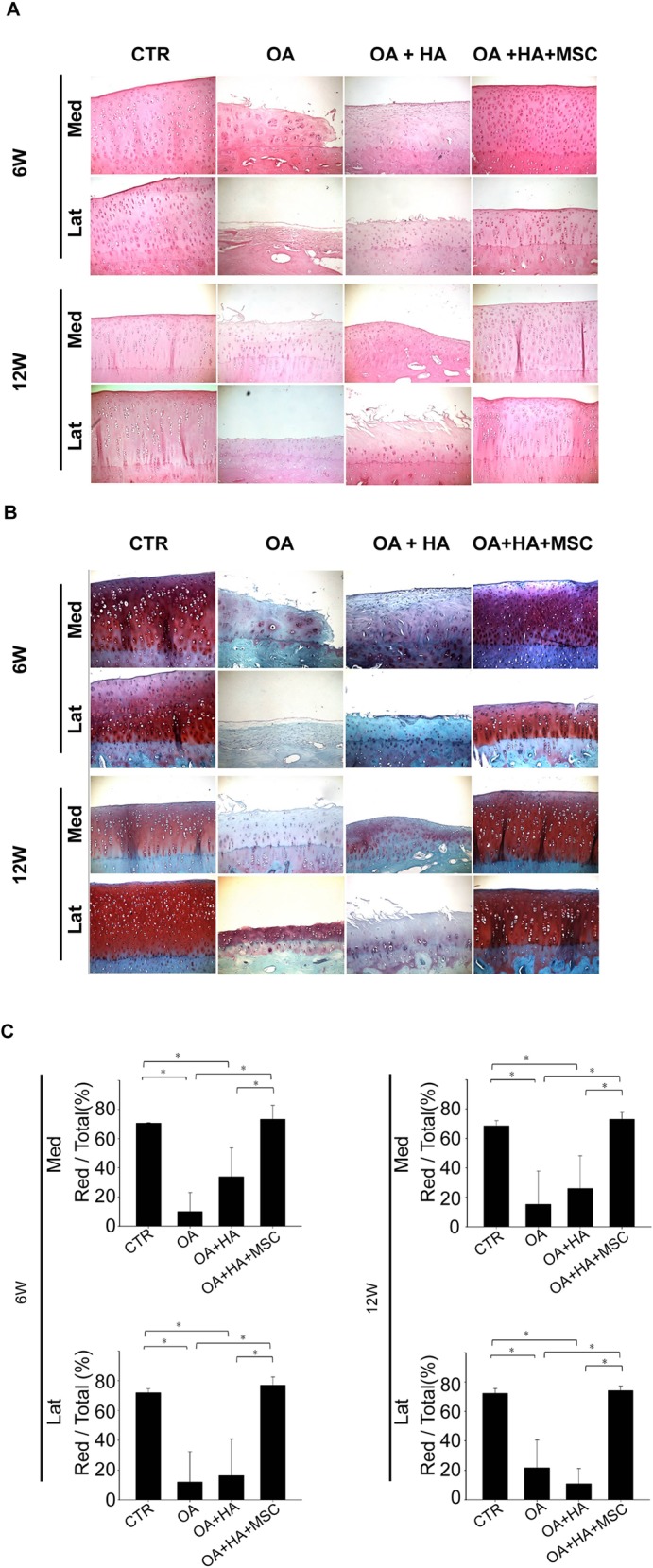
Histologic analysis. **(A, B)** Femur condyles of animals at 6 and 12 weeks post-treatment were assessed by **(A)** haematoxylin and eosin and **(B)** Safranin-O staining. **(C)** Quantitative analysis of safranin-O staining. Data are mean ± standard deviation. *P <0.05.

Representative photomicrographs of HE-stained articular cartilage sections from the OA joints and the contralateral control joints (sham), as well as those from the OA+HA and the contralateral OA+HA+MSC groups are shown in Fig 3A. Knee joints of rabbits receiving ACLT showed surface irregularity, fibrillation or cleft, changes in cellularity, and loss of tidemark integrity, while the knee joints of the sham group were devoid of these features of OA (Fig 3A). Less cartilage loss and surface irregularity in the medial and lateral compartments were noted in joints treated with HA+MSCs as compared to the OA and OA+HA groups (Fig 3A). Moreover, there was no significant difference between OA and OA+HA groups (Fig 3A), suggesting HA alone did not show therapeutic effects in OA in terms of cartilage loss and surface irregularity.

Representative photomicrographs of Safranin O-stained articular cartilage sections from the knee joints of each group were shown in Fig 3B. The ratio of Safranin O-stained area to total area (red:total) was compared (Fig 3C). Whereas the knee joints of rabbits receiving ACLT showed a reduction in Safranin O staining, those of the sham group were devoid of proteoglycan loss (Fig 3A). Analysis of the Safranin-O staining revealed a significant loss of proteoglycan in the OA and OA+HA groups, while no proteoglycan loss was noted in OA+HA+MSCs (Fig 3B). Moreover, there was no difference between OA and OA+HA groups (Fig 3B), suggesting HA alone did not show therapeutic effects in OA in terms of proteoglycan loss.

**Fig 4:**
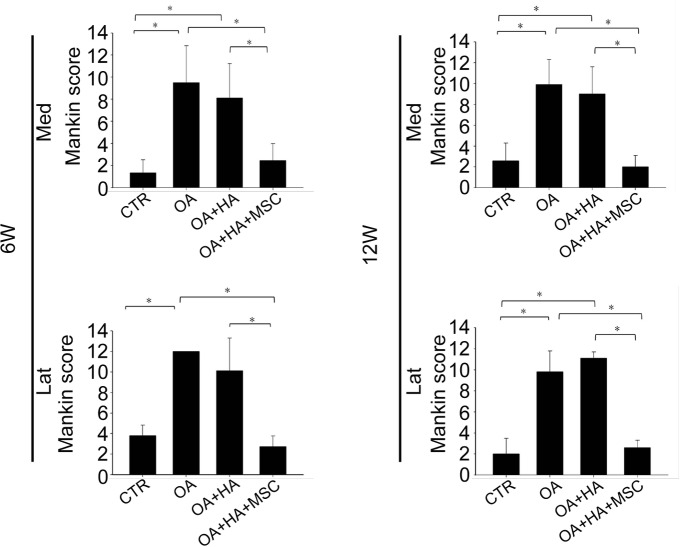
Histological grading of the effect of hypoxia-cultured MSCs. Mankin scores at different part of the joint at 6 and 12 weeks post-treatment. Data are mean ± standard deviation. *P <0.05.

The modified Mankin score showed a significant increase in OA and OA+HA when compared to the shame (Fig 4). There was no difference between OA and OA+HA, suggesting HA alone did not improve OA. Moreover, the modified Mankin score was significantly reduced in OA+HA+MSCs when compared to that of OA and OA+HA. These reductions of modified Mankin score were consistent in different compartments of the joint at both 6 and 12 weeks post-treatment (Fig 4). These data suggest HA plus MSCs rather than HA alone is effective in suppressing OA histological changes.

### Immunohistochemistry staining for type II and type X collagen

**Fig 5:**
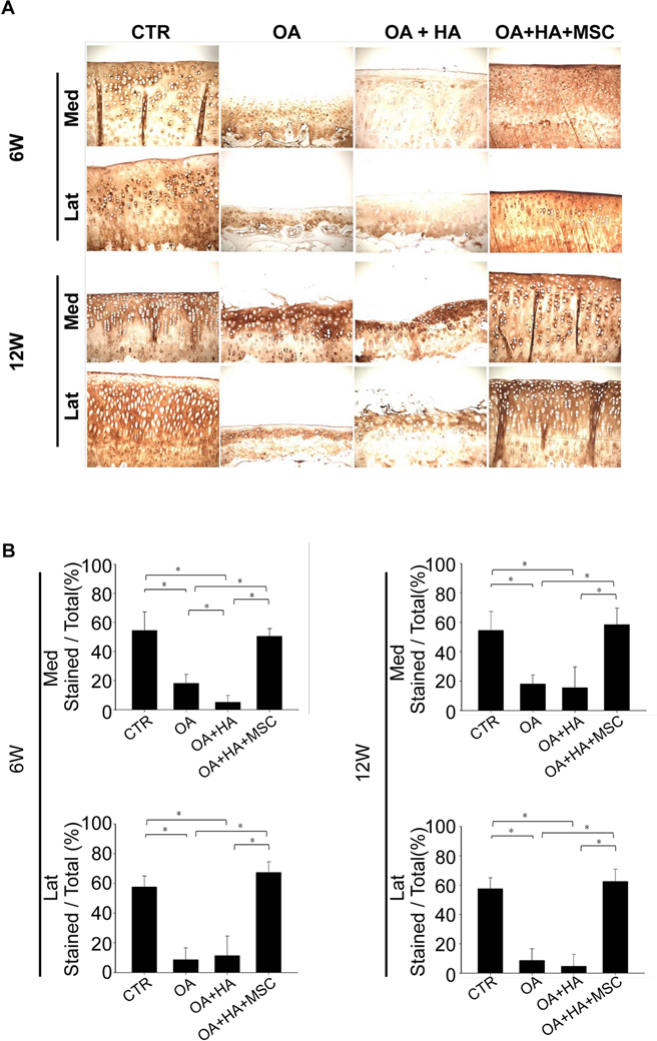
Immunohistochemistry analysis of type II collagen. **(A)** Femur condyles stained with type II collagen (Magnification, ×100). **(B)** Quantitative analysis of type II collagen. Data are mean ± standard deviation. *P <0.05.

Representative photomicrographs of the immunohistochemistry analysis of the articular cartilage from all four groups are shown in Fig 5A. As shown in Fig 5B, the density of immunolocalized type II collagen in the OA or OA+HA groups was significantly smaller than that of the shame and OA+HA+MSCs groups at both 6 and 12 weeks after treatment. Moreover, the density of immunolocalized type II collagen in the OA+HA group is not greater than that of the OA group at both 6 and 12 weeks after treatment (Fig 5B).

**Fig 6:**
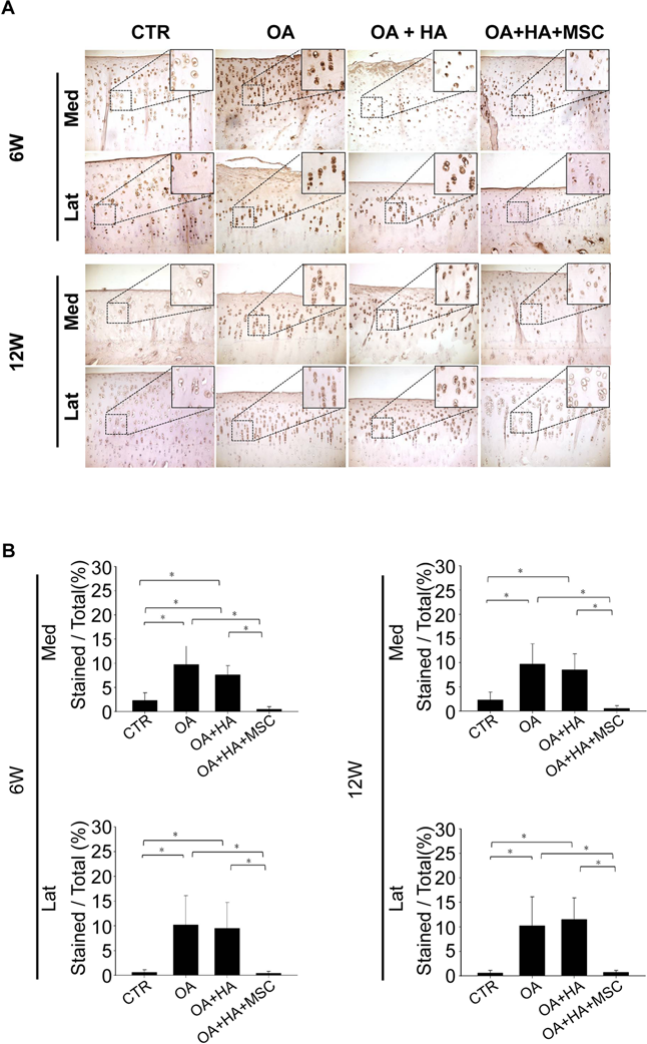
Immunohistochemistry analysis of type X collagen. **(A)** Microscopic appearance of femur condyles stained with type X collagen (Magnification, ×100; Upper box, ×400). **(B)** Quantitative analysis of type X collagen. Data are mean ± standard deviation. *P <0.05.

Immunolocalized type X collagen was predominantly found in the articular chondrocytes from the OA or OA+HA groups at both 6 and 12 weeks (Fig 6A). There was no difference in the density of immunolocalized type X collagen between OA and OA+HA groups (Fig 6B). Moreover, immunolocalized type X collagen was less evident in the cartilage joints treated with HA plus MSCs as compared with those treated with HA alone at both 6 and 12 weeks (Fig 6A and 6B). These data demonstrate the superior effect of HA plus MSCs in decreasing the density of immunolocalized type X collagen compared with HA alone. A significant increase in peri-chondrocyte staining for type X collagen was also noted in the OA and OA+HA groups as compared to OA+HA+MSCs group (Fig 6A, inserts). Together, these data suggest the inhibitory effect of MSCs on the production of type X collagen.

### *In vivo* localization of hypoxic-preconditioned MSCs

**Fig 7:**
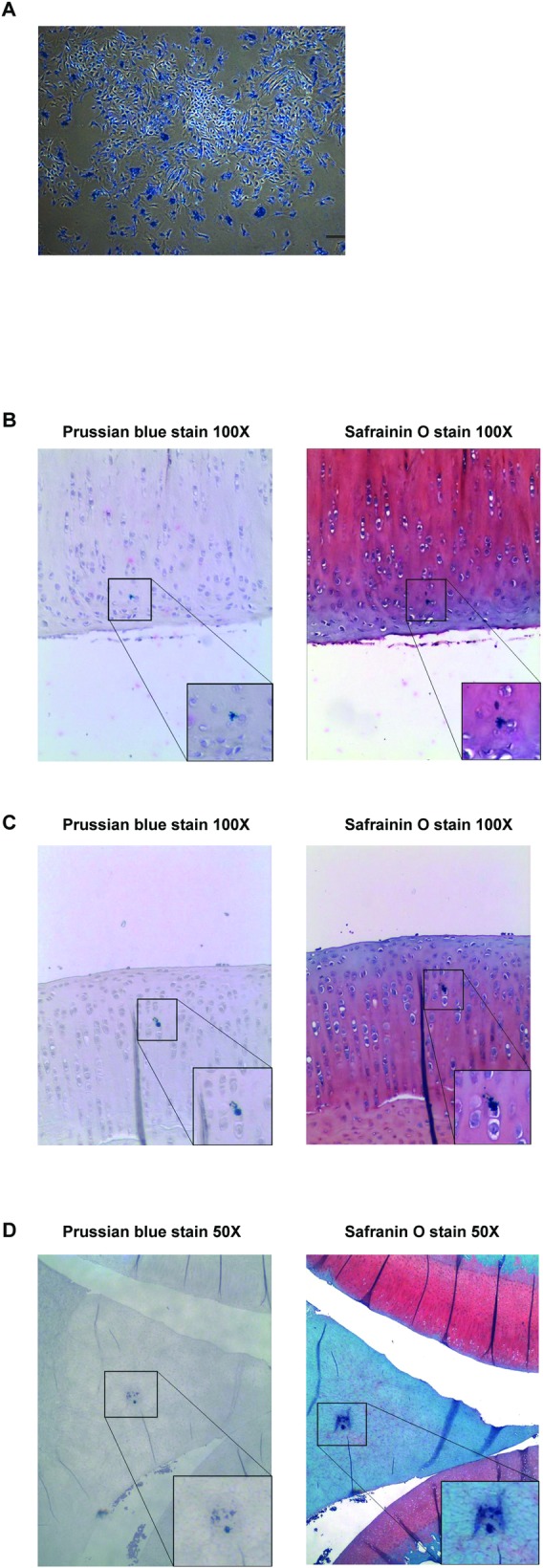
Identification and localization of SPIO-labeled MSCs. **(A)** Endocytosis of SPIO nanoparticles by MSCs visualized by Prussian blue staining (bar = 100 μm). **(B-D)** Engraftment of injected MSCs into cartilage of the (B) femoral condyle (Magnification, ×100), (C) tibial plateau (Magnification, ×100) and (D) meniscus (Magnification, ×50) by Prussian blue and Safranin-O staining. (Lower boxes, (B, C) ×200, (D) ×100).

Prussian blue staining confirmed the high efficiency of endocytosis of SPIO nanoparticles by the MSCs (Fig 7A). Scattered SPIO-labeled MSCs were identified throughout the knee joint *in vivo* specifically in the cartilage of femur (Fig 7B), tibia (Fig 7C) and meniscus (Fig 7D). These findings suggest that SPIO-labeled cells migrated into the surface of the cartilage and scattered in different parts of the joint 12 weeks after MSC injection.

## Discussion

In this study, intra-articular injection of HA combined with allogeneic, MSCs could significantly prevent OA progression in an *in vivo* ACLT model of OA in rabbits. This effect was evident by reduced osteophyte formation, cartilage wearing, and subchondral bone exposure, as well as better extracellular matrix milieu. In contrast, HA alone did not provide any therapeutic benefits in inhibiting OA progress. The positive impact of MSC treatment was observed in both the medial and lateral compartments of the knee joint as early as 6 weeks post-MSC injection and was maintained after 12 weeks. Scattered MSC engraftment was also observed throughout the joint, including within the femoral and tibial cartilage surface, meniscus and synovium.

Treatment of diffuse chondral lesions largely depends on surgical intervention, such as arthroscopic debridement or knee replacement. Alternatively, direct intra-articular injection of MSCs may represent the simplest approach to this clinical scenario. In a caprine model of OA, Murphy et al. [17] injected autologous bone marrow-derived MSCs and observed reduced signs of OA and marked regeneration of the medial meniscus. Similarly, in a collagenase-induced OA model, Huurne et al. [18] showed that early-stage injection of autologous adipose-derived MSCs inhibited synovial thickening and cartilage destruction. In another study, Sato et al. [19] showed that partial cartilage repair with strong immunostaining for type II collagen in human MSC-treated guinea pig spontaneous OA. Furthermore, Toghraie et al. [20] demonstrated that injection of fat pad-derived allogenic MSCs resulted in less osteophyte formation and subchondral sclerosis in rabbits with ACLT-induced OA; however, this treatment was not effective until 20 weeks of injection. In the present study, we observed that injection of bone marrow-derived allogeneic MSCs significantly suppressed osteoarthritic-induced changes in ACLT-induced OA while markedly increasing the content of type II collagen and reducing peri-chondrocyte type X collagen production, indicating that not only the structure but also the content of the MSC-treated cartilage was significantly improved. Furthermore, this positive effect was observed as early as 6 weeks.

Intra-articular HA injection has been utilized for treating OA but has yielded inconsistent clinical effect [21]. Similarly, in a systemic review by Edouard et al. [22], repair of articular cartilage following HA injection was observed among different animal models. In the current study, no significant effect of HA injection was observed as compared to non-treated OA model. This ineffectiveness might be due to sub-optimal dosage of HA for cartilage repair, single-dose injection or the prolonged interval between OA induction and HA injection. HA has been identified as a crucial modulator in many physiological and pathological processes in cartilage through its interaction with CD44 on the surface of cells such as chondrocytes [23;24]. Moreover, HA coats articular cartilage surface, locating near the collagen fibrils and sulfated proteoglycans within the cartilage [25;26]. We took advantage of this interaction in the hope that it would serve as a vehicle for delivering the MSCs to cartilage, and engraftment of these hypoxia-cultured MSCs was evident throughout the joint in different parts of the cartilage surface. However, the optimal proportion of HA to MSCs for engraftment remains to be elucidated. Besides serving as a vehicle for delivering MSCs, HA has potential biological effects, including the enhancement of the chondrogenic effects of MSCs [27] and the promotion of synovial cell or chondrocyte migration in the presence of basic fibroblast growth factors [28].

Homing and survival or engraftment of transplanted cells play a key role in sustaining the therapeutic effects of cell transplantation [9;29]. Engraftment of injected MSCs into diffuse osteoarthritic cartilage showed mixed results. Murphy et al. [17] observed that the engraftment of MSCs was located only in the synovial capsule, fat pad and lateral meniscus, but not in joint cartilage. In contrast, Sato et al. showed that injected MSCs adhered to the surface and scattered within the cartilage 5 weeks post-injection [19]. In one collagenase-induced murine OA model, transplanted MSCs were located in the cruciate ligament area and within subintimal layer of synovium 24 h after injection; however, they were no longer detected at 5 days post-injection [18]. In the present study, MSCs were identified 12 weeks after injection, implying that these cells might exert a long-lasting effect after injection. Notably, there are several differences among these studies. First, different animals with different OA models were chosen to evaluate the therapeutic effects of MSCs on OA. Second, autologous or allogeneic MSCs from different species and different culture conditions were investigated. Third, some studies injected MSCs in combination with HA [17;19] but some did not [18]. We chose posttraumatic OA model and investigated the therapeutic effects of HA plus allogenic rabbit bone marrow derived MSCs expanded under hypoxia. Thus, it is difficult to make a conclusion that hypoxia-expanded MSCs engrafted longer than normoxia-expanded MSCs and had a better lasting effect on OA treatment. Future side by side comparison of the therapeutic effects of hypoxia- and normoxia-expanded MSCs in the same OA models is required to answer this question. Moreover, further efforts are also required to focus on the cell fate and proliferation status of transplanted cells and the potential immune issues encountered upon allogeneic transplantation of MSCs.

In summary, injection of allogeneic MSCs plus HA suppresses OA progression in the knee joint of mature rabbits. This chondroprotective effect could be observed as early as 6 weeks after treatment at which time the MSCs were well-engrafted into both femoral and tibial cartilage. The findings presented here may be utilized as a basis for further clinical studies.

## Supporting Information

S1 Fig Differentiation potential of hypoxia-cultured bone marrow-derived MSCs. **(A)** Adipogenic differentiation. Micrographs showing Oil Red O staining at 21 days of induction; bars = 50 μm. **(B)** Osteogenic differentiation. Micrographs showing Alizarin Red S staining after 21 days of induction; bars = 100 μm. **(C)** Chondrogenic differentiation. Micrographs showing Alcian blue staining of the pellet after 21 days of induction; bars = 1 mm.
(TIF)
