# Supplementary figures and images for: Allogeneic Mesenchymal Stem Cells in Combination with Hyaluronic Acid for the Treatment of Osteoarthritis in Rabbits
Source: PLoS One. 2016 Feb 25;11(2):e0149835. doi: 10.1371/journal.pone.0149835 (PMC4767225; doi:10.1371/journal.pone.0149835)

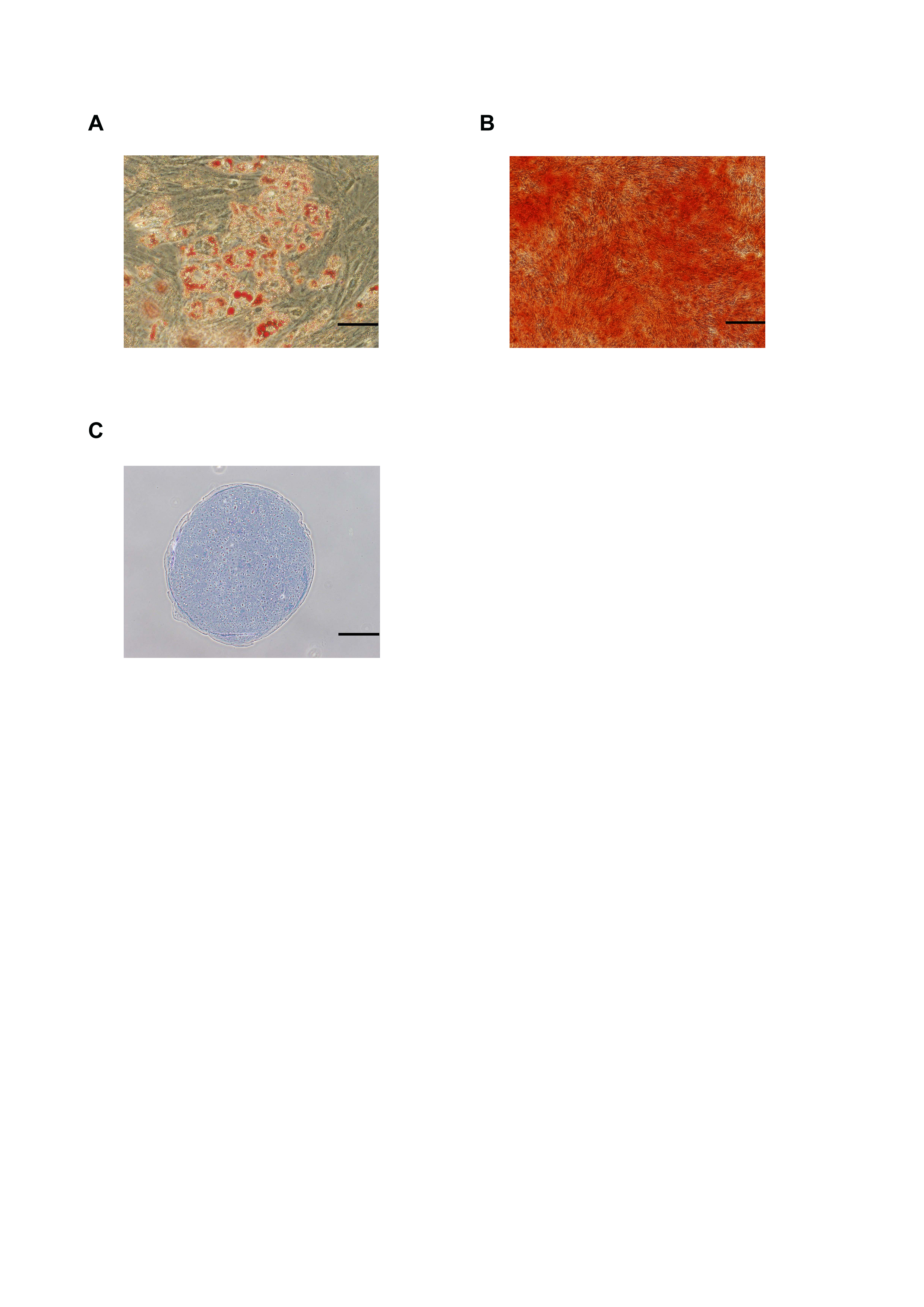

Supplement: S1 Fig — (A) Adipogenic differentiation. Micrographs showing Oil Red O staining at 21 days of induction; bars = 50 μm. (B) Osteogenic differentiation. Micrographs showing Alizarin Red S staining after 21 days of induction; bars = 100 μm. (C) Chondrogenic differentiation. Micrographs showing Alcian blue staining of the pellet after 21 days of induction; bars = 1 mm. (TIF) [file pone.0149835.s001.tif]
